# Application of Population Sequencing (POPSEQ) for Ordering and Imputing Genotyping-by-Sequencing Markers in Hexaploid Wheat

**DOI:** 10.1534/g3.115.020362

**Published:** 2015-10-29

**Authors:** Erena A. Edae, Robert L. Bowden, Jesse Poland

**Affiliations:** * USDA-ARS, Hard Winter Wheat Genetics Research Unit, Manhattan, Kansas 66506; † Wheat Genetics Resource Center, Department of Plant Pathology and Department of Agronomy, Kansas State University, Manhattan, Kansas 66506

**Keywords:** GBS, WGS, POPSEQ, linkage map, imputation

## Abstract

The advancement of next-generation sequencing technologies in conjunction with new bioinformatics tools enabled fine-tuning of sequence-based, high-resolution mapping strategies for complex genomes. Although genotyping-by-sequencing (GBS) provides a large number of markers, its application for association mapping and genomics-assisted breeding is limited by a large proportion of missing data per marker. For species with a reference genomic sequence, markers can be ordered on the physical map. However, in the absence of reference marker order, the use and imputation of GBS markers is challenging. Here, we demonstrate how the population sequencing (POPSEQ) approach can be used to provide marker context for GBS in wheat. The utility of a POPSEQ-based genetic map as a reference map to create genetically ordered markers on a chromosome for hexaploid wheat was validated by constructing an independent *de novo* linkage map of GBS markers from a Synthetic W7984 × Opata M85 recombinant inbred line (SynOpRIL) population. The results indicated that there is strong agreement between the independent *de novo* linkage map and the POPSEQ mapping approach in mapping and ordering GBS markers for hexaploid wheat. After ordering, a large number of GBS markers were imputed, thus providing a high-quality reference map that can be used for QTL mapping for different traits. The POPSEQ-based reference map and whole-genome sequence assemblies are valuable resources that can be used to order GBS markers and enable the application of highly accurate imputation methods to leverage the application GBS markers in wheat.

Bread wheat (*Triticum aestivum* L.) is one of the world’s most important cereal crops, providing approximately 20% of all calories consumed by humans, and is a staple food crop for 30% of the human population. Increasing wheat production at the global scale is key to the efforts of filling the anticipated future food shortage gap attributable to global population increase and adverse effects of climate change on crop production. The application of advanced and precise molecular tools is needed to speed the development of new wheat varieties.

The advent of next-generation sequencing technologies led to the emergence of high-throughput, sequence-based genotyping (Baird *et al.* 2008) approaches such as genotyping-by-sequencing (Poland and Rife 2012; Mascher *et al.* 2013). It also enabled the use of whole-genome shotgun sequencing (WGS) to generate genome assemblies of large and complex genomes. Although WGS assemblies of these large and complex genomes often contain many small, unordered contigs, assemblies can be made rapidly at low cost compared with approaches based on physical maps (Mascher *et al.* 2013; Mascher and Stein 2014).

The “gold-standard” method of genome sequencing is characterized by developing a physical map followed by sequencing of the minimum tiling path. Although the former needs coordinated efforts of several research laboratories and requires considerable investment in terms of time and money especially for large genome species like wheat, it is still considered as the indispensable method that leads toward a reference genome of a crop species without a reference genome (Feuillet *et al.* 2011, 2012). However, as a more tractable working assembly, the low-copy gene-space from the WGS approach is becoming an important genomic resource for gene discovery and functional genomics in species without a reference genome. This application has been proved on large genome crop species such as barley (*Hordeum vulgare*) and wheat by combining *de novo* WGS with classical genetic analysis (Mascher *et al.* 2013; Hahn *et al.* 2014; Chapman *et al.* 2015).

Population sequencing methodology, known as POPSEQ, was proposed as an integrated method to order and link contigs in WGS genome assemblies for gene isolation, genomics-assisted breeding, and genetic diversity assessment (Mascher *et al.* 2013; Ariyadasa *et al.* 2014). The method relies on the genetic segregation in biparental populations to create a linear order of contigs on an individual chromosome. The potential of POPSEQ to bring contigs into a linkage map was demonstrated on barley (Mascher *et al.* 2013) and then on hexaploid wheat (Chapman *et al.* 2015). A similar approach, which was referred to as recombinant population genome construction, was used to produce a high-quality genome assembly using a segregating population of *Caenorhabditis elegans* (Hahn *et al.* 2014).

Next-generation, sequencing-based genotyping approaches have been applied to understand the biological basis of agronomic traits in several plant species and for making selections via whole-genome prediction (Berkman *et al.* 2012). Studies on major crop species such as *Zea mays* (Bernardo and Yu 2007; Bernardo 2009; Crossa *et al.* 2013), *T. aestivum* (Heffner *et al.* 2010; Poland *et al.* 2012b; Daetwyler *et al.* 2014), *Oryza sativa* (Xu *et al.* 2014), and *H. vulgare* (Iwata and Jannink 2011) have indicated that genomic selection has the advantage of reducing the time needed to release new cultivars for production in plant breeding. Because genome-wide prediction needs a large number of evenly distributed markers per chromosome, genotyping-by-sequencing (GBS) markers are suitable for accurately predicting breeding values of candidate individuals in plant breeding programs. The GBS platform provides tens of thousands of markers with relatively low investment (Elshire *et al.* 2011; Poland *et al.* 2012a) and allows marker discovery and genotyping to be conducted simultaneously. GBS has been shown as an effective marker platform for whole-genome profiling and subsequent trait prediction (Poland *et al.* 2012b; Crossa *et al.* 2013; Zhang *et al.* 2014). Another promising area of application of GBS markers in plant breeding is for quantitative trait locus (QTL) mapping either by narrowing previously detected gene/QTL candidate regions or for the identification of novel gene/QTL regions that underlay economically important traits through saturating chromosomes with high-density GBS markers.

The recent availability of a draft genome sequence and gene space assemblies for hexaploid and diploid wheat also makes the GBS platform an attractive approach for gene identification and precisely mapping QTL through anchoring trait-associated GBS single-nucleotide polymorphism (SNP) tags on draft genome sequence and/or gene space assemblies. From recent QTL studies with GBS markers, saturating chromosome regions with markers enabled the detection of previously detected and novel QTL for aluminum tolerance and leaf width in rice (Spindel *et al.* 2013), for drought tolerance in chickpea (Jaganathan *et al.* 2014), and for precise identification of the location of a dwarfing gene called *Breviaristatum-e* in barley (Liu *et al.* 2014).

A drawback of genotypic data from the GBS platform is often a large proportion of missing data points across samples as genomic DNA fragments are sequenced at low depth (Elshire *et al.* 2011). For GBS marker application in genomic selection, methods of imputing missing data points for unordered markers have been developed (Rutkoski *et al.* 2013). However, many other imputation approaches that have been developed first require markers to be linearly ordered on a chromosome. Here we demonstrate the utility of the POPSEQ methodology for mapping, ordering and imputing marker data from GBS.

## Materials and Methods

### Germplasm and genotyping

We used recombinant inbred lines (RILs) from a cross between Synthetic W7984 and Opata M85 (“SynOpRIL”; Sorrells *et al.* 2011) for this study. The total number of inbred lines used in this study was 183 lines, a subset of the larger population of ∼2000 lines. Genomic DNA was extracted from seedlings of each individual line grown in a greenhouse. The GBS libraries were constructed in 96-plex following the two-enzyme system GBS protocol with restriction enzymes *PstI* (CTGCAG) and *MspI* (CCGG) (Poland *et al.* 2012a). Each library was sequenced on the Illumina HiSequation 2000 platform.

### SNP calling and sequence data processing

Raw sequence data were processed with a custom java script in TASSEL 4, and a population-based SNP calling approach was used (Poland *et al.* 2012a). Unique sequence tags of 64 bp were aligned internally allowing mismatches of up to 3 bp to identify SNPs within the tags. Fisher’s exact test was applied to determine independence of SNP alleles and then filter the SNPs. SNPs with up to 80% missing data points were retained for subsequent data analysis.

### Linkage map construction and comparison with POPSEQ data

Construction of a linkage map for Synthetic W7984 × Opata M85 RIL population was performed with MSTMap software (Wu *et al.* 2008) to group the markers into linkage groups. A total of 6362 polymorphic markers with up to 20% missing data, minor allele frequency of greater than 30%, and heterozygosity of less than 2% were considered for linkage map construction. Logarithm of odds score of 10 was used to cluster the markers into linkage groups. Linkage groups from the same chromosome were merged together and markers of the same chromosome were reordered with MSTmap.

We used the synthetic wheat W7984 and Chinese Spring shotgun assemblies that were ordered with the POPSEQ approach using Synthetic W7984 × Opata M85 doubled haploid population (SynOpDH) to anchor SNP tags on genetically ordered WGS contigs (The International Wheat Genome Sequencing Consortium 2014; Chapman *et al.* 2015). To summarize in brief, the SynOpDH population, consisting of 90 individuals, was shotgun-sequenced, and SNPs were identified to construct a high-density linkage map that has all 21 wheat chromosomes. Then this highly dense genetic map was used to linearly order SNP-associated contigs in the WGS assembly of W7984 (Chapman *et al.* 2015). The contigs of Chinese Spring assembly were also integrated into the same map of SynOpDH population (The International Wheat Genome Sequencing Consortium 2014).

Tags from which we detected SNPs were first aligned against both W7984 and Chinese Spring assemblies. To assemble the SNP tags, we used bwa software with “aln” method with default set up. The Samtools “view” method was used to further process sequence output. The SNP tags were filtered with a minimum alignment quality score of 37, and then markers that passed the quality requirements were merged with the high-density genetic map from SynOpDH population. Both W7984 and Chinese Spring assemblies and the high-density genetic map were linked by scaffold name in the former and by contig name in the latter, which facilitated navigation from assembly to genetic map or vice versa.

Independently constructed *de novo* linkage maps were compared with the POPSEQ-based map for the number of markers correctly assigned to their respective linkage groups, and the linear relationship of marker order between *de novo* and POPSEQ maps. The step-by-step procedure of integrating GBS tags into the POPSEQ assembly is indicated in Supporting Information, Figure S1.

### Missing data imputation

Genotypic data imputation was performed after anchoring SNP tags to the POPSEQ assembly. We aligned sequences of SNP tags for 33,664 SNPs that were obtained from the SynOpRIL population to W7984 and Chinese Spring assemblies to impute missing data points. For W7984 assembly, a total of 16,591 markers, and for Chinese Spring 9709 markers were imputed by use of the FSFHap (Full-Sib Haplotype imputation) method implemented in TASSEL 5 (Swarts *et al.* 2014). The algorithm first detects two parental haplotypes and recombination break points via a Hidden Markov model and Viterbi algorithm. A missing data point is imputed to the matching genotype of flanking markers. If genotypes of markers flanking a missing data point do not match, the missing data point is left missing.

Imputation accuracy of FSFHap method also was assessed by the use of squared correlation coefficient (R^2^). To calculate this parameter, 5% of the total genotypes were masked randomly for each marker in the dataset at genotypes where the SNP call was present. Squared correlation coefficient (R^2^) was calculated by comparing the original data where SNP calls were masked with corresponding imputed data for each marker. The R^2^ calculation also was performed for the markers common between Chinese Spring and W7984 assemblies.

### Data availability

Recombinant Inbred Lines used in this study are available on request from the Wheat Genetic Resource Center at KSU. Sequence data is available from NCBI SRA under SRP066497.

## Results

### Validating POPSEQ with *de novo* linkage map

**Figure 1:**
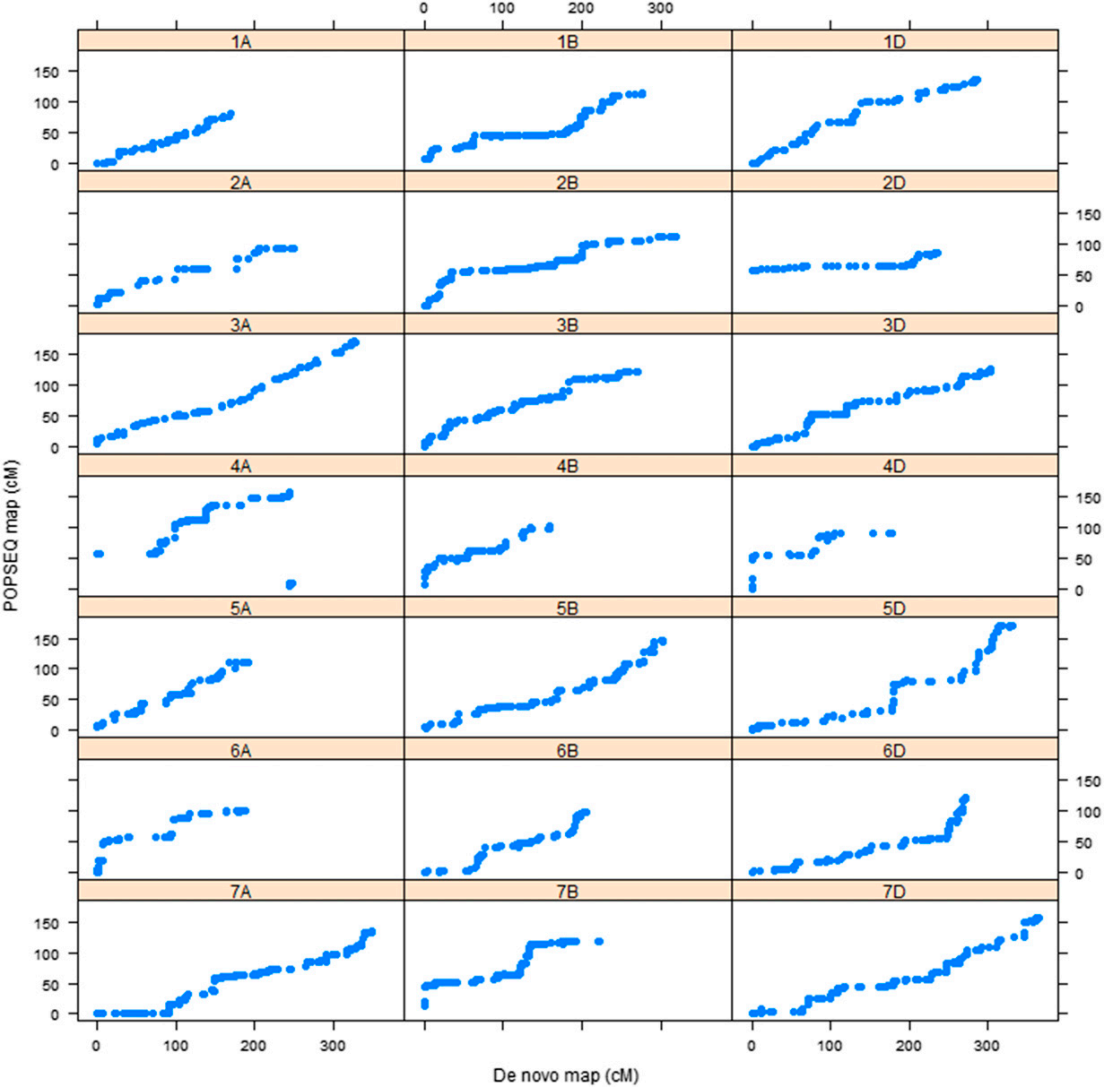
Marker order relationship between *de novo* map and POPSEQ map for all 21 chromosomes of hexaploid wheat for markers anchored to W7984 assembly.

**Figure 2:**
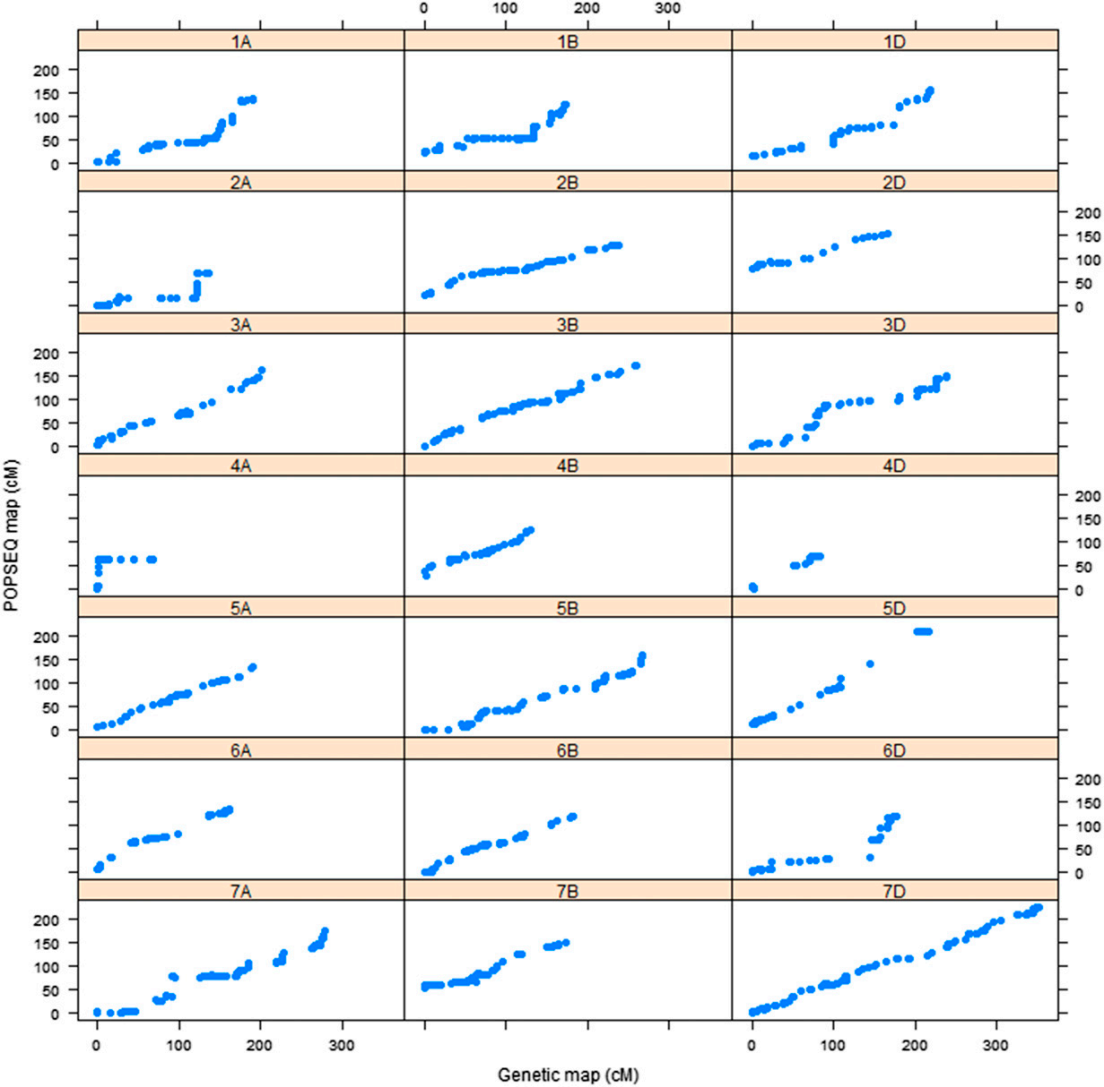
Marker order relationship between *de novo* map and POPSEQ map for all 21 chromosomes of hexaploid wheat for markers anchored to Chinese Spring assembly.

The use of an ultradense genetic linkage map constructed from the 90-individual doubled haploid population (SynOpDH) with the POPSEQ approach was validated through integrating SNP tags of GBS markers used to construct *de novo* linkage maps. We used an independent RIL mapping population made from the same synthetic W7984 and Opata M85 parents for this validation work. A total of 23 linkage groups with greater than two markers were obtained with a logarithm of odds score of 10. There was a total of 6360 markers in the linkage groups. Aligning sequences of SNP tags of these markers with sequences of linearly ordered gene space assembly of W7984 and Chinese Spring resulted in 3364 markers and 2049 markers that passed minimum quality alignment score of 37, respectively. A total of 3357 (99.8%) for W7984 and a total of 2037 markers (99.4%) for Chinese Spring were assigned correctly to their respective linkage group based on the sequence identity search in the assemblies. After removing incorrectly assigned markers and markers reported suspicious by the mapping algorithm in MSTmap, 21 linkage groups that correspond to the total number of hexaploid wheat chromosomes were obtained. Comparison of genetic map positions of the GBS markers in each of the 21 linkage groups based on the *de novo* map with that of positions of each marker based on the high density genetic map of POPSEQ also showed that there was a linear relationship between the two genetic maps (Figure 1 and Figure 2).

**Figure 3:**
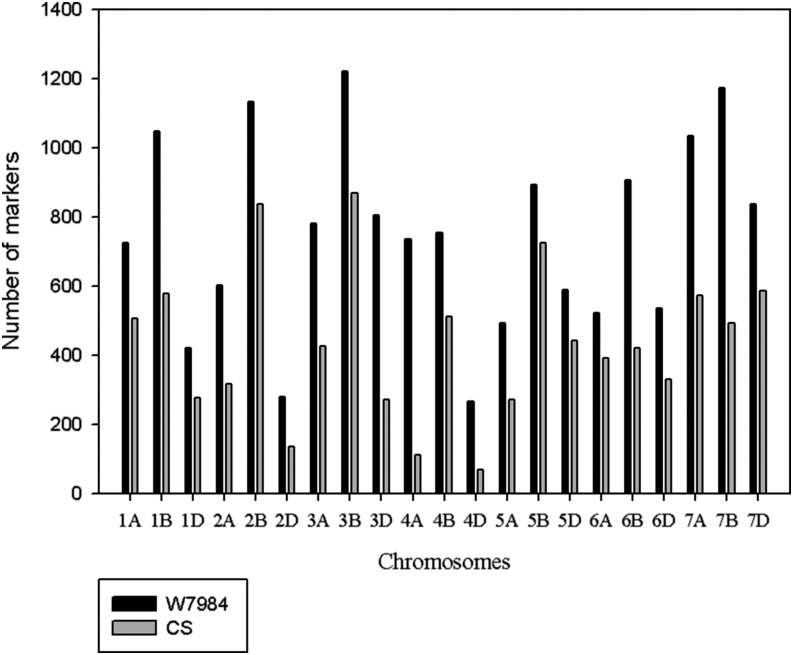
Genome-wide distribution of genotype-by-sequencing markers anchored to both W7984 and Chinese Spring (CS) assemblies.

**Figure 4:**
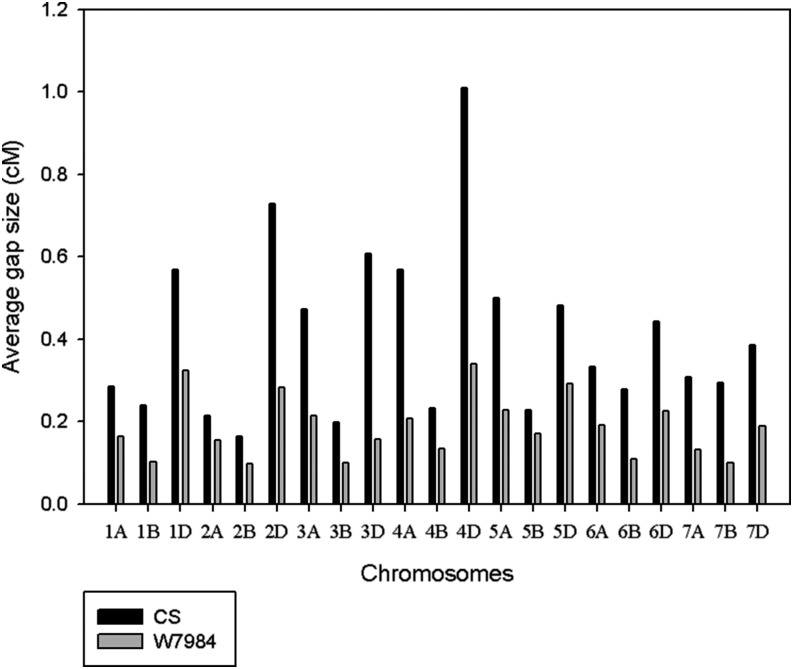
Average gap size (cM) for the markers anchored to both W7984 and Chinese Spring (CS) assemblies.

Originally, a total of 33,664 GBS markers with up to 80% missing data were obtained for the synthetic W7984 × Opata M85 RIL population. All marker tags, without consideration of the level of missing data, were integrated into POPSEQ data. After discarding markers with low alignment quality score (<37), we found map positions for 16,591 markers (49.3%) for W7984 assembly and 9709 (28.8%) for Chinese Spring assembly. The number of markers per chromosome was greater for W7984 assembly for all chromosomes than that of Chinese Spring (Figure 3 and Table S1). Similarly, the average gap size per chromosome was low for all chromosomes in the case of W7984 assembly (Figure 4). Maximum gap size for markers anchored to W7984 assembly was lower than 20 cM for all chromosomes. All chromosomes had less than 30 cM maximum gap size for markers anchored to the Chinese Spring assembly (Table S2). The two assemblies had a total of 7040 markers in common, and there was a strong positive relationship between marker and map positions between the two maps (Figure S2).

### Data imputation

Two datasets comprised a total of 16,591 markers (for W7984 assembly) and 9709 markers (for Chinese Spring) were submitted to FSFHap for imputation. After imputation, 95% of the markers for W7984 assembly and 94% of the markers for Chinese Spring had less than 10% missing data points (Table S3 and Table S4). Furthermore, 92.4% and 89.8% of the markers had less than 5% of missing data for W7984 assembly and Chinese Spring assembly, respectively. Only 2.7% of the markers for W7984 assembly and 3.2% of the markers for Chinese Spring assembly had more than 20% remaining missing data points per marker. The greatest average missing data points per chromosome in the imputed data set was recorded for chromosome 2D (Figure S3), and there was no clear relationship between the amount of missing data per chromosome in the original and imputed data sets (Figure S4 and Figure S5). The number of un-imputed missing data points was greater for Chinese Spring than that of W7984 assembly in 15 chromosomes. The average proportion of heterozygous genotypes per marker was comparable between the two assemblies except for chromosomes 2A, 3D, and 4A, where differences between the two assemblies were large (Figure S6). The exceptionally high average proportion of heterozygous genotypes for chromosome 2A for Chinese Spring was due to high heterozygous genotypes per marker (28–37%) for markers within the interval 58.7−67.4 cM (around the centromeric region) (Table S4). Markers on chromosome 4D had high heterozygote genotypes for both assemblies. However, except for these two chromosomes (2A and 4D), the average proportion of heterozygote genotypes per marker was less than 10% for all chromosomes (Figure S6). There was no relationship between the amount of heterozygote genotypes before imputation and the amount remaining after imputation for both assemblies (Figure S7 and Figure S8). The average proportion of heterozygote genotypes per markers in imputed data set was greater than that of before imputation for both assemblies for all chromosomes. The algorithm implemented in FSFHap is taking into account heterozygote undercalling for GBS platform SNP calling. However, taking into account the low proportion of heterozygote genotypes in the original data and the expected residual heterozygosity for RIL populations, FSFHap may be overestimating the amount of heterozygote genotypes per marker in the imputed data.

Imputation accuracy of FSFHap was evaluated by the use of squared correlation coefficient (R^2^). The average R^2^ for the markers anchored to Chinese Spring was 0.94, and 90% of the markers had R^2^ greater than or equal to 0.8 (Figure S9). Similar average R^2^ of 0.94 was obtained for the markers common between Chinese Spring and W7984 assemblies.

## Discussion

We validated the utility of POPSEQ, a reference map-based marker ordering method, by using a new RIL population. We first constructed a *de novo* genetic linkage map by using filtered, high-quality GBS markers from the Synthetic W7984 × Opata M85 RIL population. For the POPSEQ map to be used as a reference map to order the GBS markers from different populations, first GBS markers within a *de novo* linkage group should be assigned to the same chromosome with the chromosome assignment of the markers based on aligning SNP tags sequences with the POPSEQ genome assembly. Second, marker order of the *de novo* linkage map should also agree with the order of the same markers on the POPSEQ-based reference genetic map. When a high alignment stringency level was used, both chromosome assignment and marker order indicated that there is a good agreement between the *de novo* linkage map and POPSEQ-based high-density genetic map. This supports that the POPSEQ approach can be used as an alternative method to assign and order GBS markers on wheat chromosomes.

The advantage of using the POPSEQ approach for mapping and ordering GBS markers is twofold. In classical linkage mapping approach, chromosome assignment information is required for all constructed linkage groups. With the absence of this information, anchor markers are needed to guide linkage map construction and for downstream interpretation of QTL analysis. In the case of GBS markers, for species without a reference genome sequence, this information is presumably lacking and consequently construction of a *de novo* linkage map is a tedious job. Moreover, genetic map construction is limited by the large number of markers with a high proportion missing data. However, the POPSEQ anchored reference can give *a priori* marker positions as with any reference genome and avoids the need of both anchor markers and *de novo* map construction.

By using the POPSEQ approach, we were able to genetically map and order 16,591 and 9711 GBS markers anchored to W7984 and Chinese Spring assemblies, respectively. The greater number of markers for W7984 assembly compared with Chinese Spring assembly may be attributable to W7984 being one of the parents of the biparental population from which SNP tags were detected. Chromosome 4D had the least number of markers whereas chromosome 3B the greatest number of markers for both assemblies (Figure 3 and Table S1). This agrees with the observation that the larger the chromosome size, the more number of markers it contains (Poland *et al.* 2012a; Saintenac *et al.* 2013). Similarly, as expected the total number of markers mapped to the D genome (25% for both assemblies) was lower than that of A (31% for W7984 assembly and 28% for Chinese Spring assemblies) and B (44% for W7984 assembly and 47% for Chinese Spring assembly) genomes as the D genome of hexaploid wheat is smaller and less diverse than A and B genomes (Chao *et al.* 2010; Wang *et al.* 2013; Edae *et al.* 2014; Iehisa *et al.* 2014). However, the difference between the number of markers mapped to D and A genomes was lower than reported in the literature, which was about fivefold greater for both A and B genomes (Allen *et al.* 2011, 2013; Cavanagh *et al.* 2013). One of the probable explanations for this small difference between A and D genomes is attributable to reintroduction of D genome diversity through synthetic W7984 to the current hexaploid wheat mapping population.

The maximum gap size per chromosome for markers anchored to W7984 assembly (<20 cM) was lower than that of markers anchored to Chinese Spring assembly (<30 cM). The original reference map from POPSEQ had good marker coverage (maximum gap size <20 cM) for all chromosomes with the exception of 1A and 4D with maximum gap size of 26 cM and 28 cM, respectively (data not shown). Overall, the pattern of marker distribution on chromosomes observed for our population also has good agreement with that of reference map, indicating the potential of POPSEQ approach of marker ordering and imputing. However, our final high-quality SNP dataset represented only approximately 30 and 49% of the total original 33,664 SNP tags for Chinese Spring and W7984 assemblies, respectively. A majority of the markers did not pass the strict sequence alignment threshold we used to reduce the risk of assigning the tags to incorrect positions. Therefore, for full utilization of the POPSEQ-based gene space assemblies, consensus reference genetic maps of two or more populations are needed.

Imputing missing data are a necessary step, particularly for the genotypic data sets with a large proportion of missing data per marker (up to 80% in our case). The high-density genetic map enabled us to use FSFHap as implemented in TASSEL 5 (Swarts *et al.* 2014). FSFHap generally needs a large data set for best results. Imputation accuracy measurements (R^2^) found here indicated that FSFHap is still accurate in imputing a relatively small dataset with a large proportion of missing data. We found average imputation accuracy (R^2^ = 0.94) roughly similar with that of average imputation accuracy (R^2^ = 0.97) obtained for a large genotypic data set of maize nested association mapping panel (NAM RILs) used by Swarts *et al.* (2014). We observed an increase in the number of heterozygous genotypes called in the imputed data. Low-coverage sequencing with GBS is expected to undercall true heterozygous genotypes. Therefore, an increase in heterozygous calls is expected to some extent with the GBS imputation. Apart from the effect of algorithms implemented in FSFHap, the greater levels of heterozygosity for some markers near centromeric regions (*e.g.*, 2A) in the imputed dataset may be the result of deleterious alleles *in trans*-linkage combined with low levels of recombination in these regions (McMullen *et al.* 2009).

In conclusion, POPSEQ methodology can be used as an alternative method of mapping and ordering GBS markers. For crop species lacking a high-quality reference genome, like hexaploid wheat, genetically ordered markers allow the implementation of marker-order−dependent but highly accurate imputation algorithms (*e.g.*, FSFHap) to impute genotypic data with a large proportion of missing data points so that QTL/gene mapping can be done precisely. Because both the Chinese Spring and W7984 POPSEQ results were based on the same SynOpDH genetic mapping population, full integration of markers to the reference map needs POPSEQ-based, high-density genetic map and gene space assemblies that are developed from additional biparental populations.

## Supplementary Material

Supporting Information
