# Supplementary material for: Application of Population Sequencing (POPSEQ) for Ordering and Imputing Genotyping-by-Sequencing Markers in Hexaploid Wheat
Source: G3 (Bethesda). 2015 Oct 29;5(12):2547–53. doi: 10.1534/g3.115.020362 (PMC4683627; doi:10.1534/g3.115.020362)
Supplement: Supporting Information [file supp_5_12_2547__index.html]

Application of Population Sequencing (POPSEQ) for Ordering and Imputing Genotyping-by-Sequencing Markers in Hexaploid Wheat — Supporting Information 

# Application of Population Sequencing (POPSEQ) for Ordering and Imputing Genotyping-by-Sequencing Markers in Hexaploid Wheat

## Supporting Information for Edae, Bowden, and Poland, 2015

**Files in this Data Supplement:**

- Figure S1 - Schematic representation of GBS marker integration into POPSEQ data. (.tif, 51 KB)
- Figure S6 - Average proportion of heterozygote genotypes after imputation for W7984 and Chinese Spring. (.tif, 717 KB)
- Figure S7 - Average proportion of heterozygote genotypes in imputed and unimputed markers anchored to W7984 assembly. (.tif, 717 KB)
- Figure S8 - Average proportion of heterozygote genotypes in imputed and unimputed markers anchored to Chinese Spring assembly. (.tif, 717 KB)
- Figure S9 - Imputation accuracy of FSFHap imputed Chinese Spring anchored markers. (.tiff, 558 KB)
- Table S1 - Number of markers anchored to both W7984 and Chinese spring for all 21 wheat chromosomes. (.xlsx, 11 KB)
- Table S2 - Maximum gap size for markers anchored to W7984 and Chinese spring assemblies for 21 wheat chromosomes. (.xlsx, 11 KB)
- Table S3 - Genotypic data of SynOPRIL population for the GBS markers anchored to W7984 assembly. (xlsx, 11,439 KB)
- Table S4 - Genotypic data of SynOPRIL population for the GBS markers anchored to Chinese Spring assembly. (xlsx, 6,934 KB)
- Figure S2 - Common markers between W7984 and Chinese Spring assemblies. (.tiff, 1,1747 KB)
- Figure S3 - Average proportion of missing data points remain after imputation for W7984 and Chinese Spring anchored markers. (.tif, 717 KB)
- Figure S4 - Average proportion of missing data points in imputed and unimputed markers anchored to W7984 assembly. (.tif, 707 KB)
- Figure S5 - Average proportion of missing data points in imputed and unimputed markers anchored to Chinese Spring assembly. (.tif, 707 KB)
