# Supplementary figures and images for: Application of Population Sequencing (POPSEQ) for Ordering and Imputing Genotyping-by-Sequencing Markers in Hexaploid Wheat
Source: G3 (Bethesda). 2015 Oct 29;5(12):2547–53. doi: 10.1534/g3.115.020362 (PMC4683627; doi:10.1534/g3.115.020362)

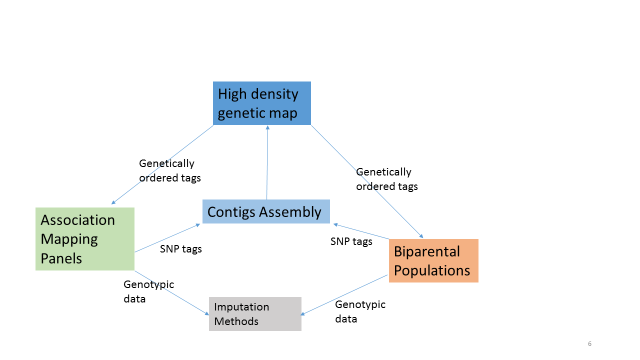

Supplement: Supporting Information [file supp_g3.115.020362_FigureS1.tif]

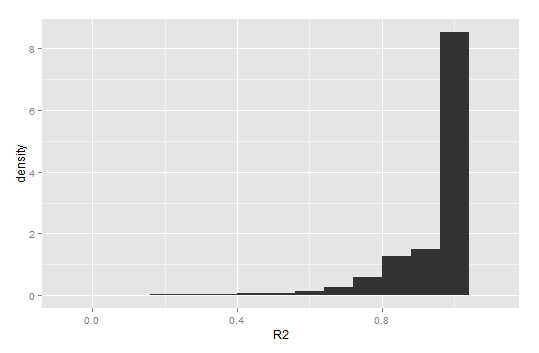

Supplement: Supporting Information [file supp_g3.115.020362_FigureS9.tif]

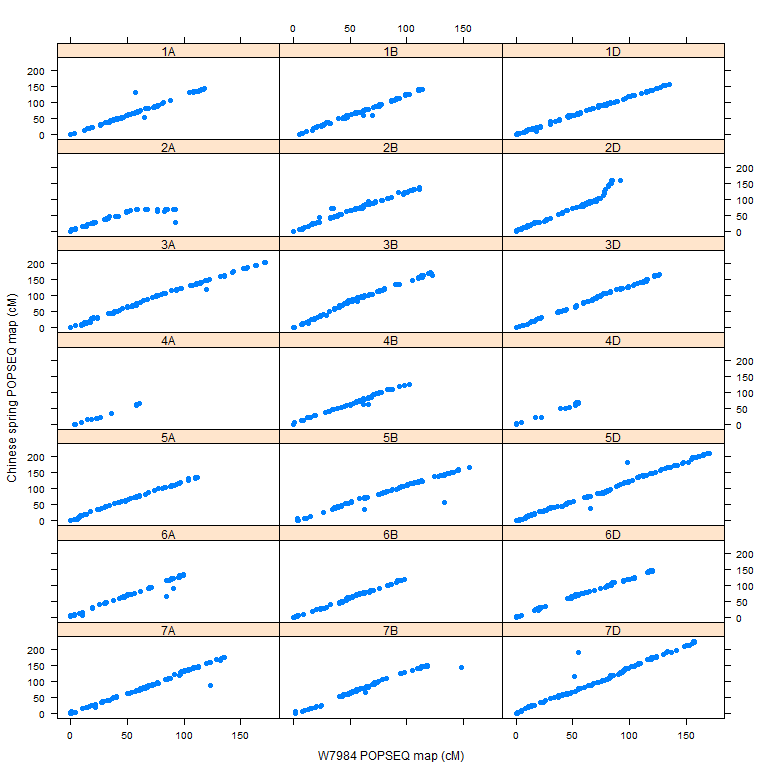

Supplement: Supporting Information [file supp_g3.115.020362_FigureS2.tif]
